# Will the real ventricular architecture please stand up?

**DOI:** 10.14814/phy2.13404

**Published:** 2017-09-26

**Authors:** Julien I. E. Hoffman

**Affiliations:** ^1^ Department of Pediatrics University of California San Francisco California

**Keywords:** Circumferential fibers, modeling, myolaminae, subendocardial left‐handed helix, subepicardial right‐handed helix, tethering

## Abstract

Ventricular twisting, essential for cardiac function, is attributed to the contraction of myocardial helical fibers. The exact relationship between ventricular anatomy and function remains to be determined, but one commonly used explanatory model is the helical ventricular myocardial band (HVMB) model of Torrent‐Guasp. This model has been successful in explaining many aspects of ventricular function, (Torrent‐Guasp et al. Eur. J. Cardiothorac. Surg., 25, 376, 2004; Buckberg et al. Eur. J. Cardiothorac. Surg., 47, 587, 2015; Buckberg et al. Eur. J. Cardiothorac. Surg. 47, 778, 2015) but the model ignores important aspects of ventricular anatomy and should probably be replaced. The purpose of this review is to compare the HVMB model with a different model (nested layers). A complication when interpreting experimental observations that relate anatomy to function is that, in the myocardium, shortening does not always imply activation and lengthening does not always imply inactivation.

## Introduction

In 1918, Arthur Keith published his Harveian Lecture, entitled “The functional anatomy of the heart” (Keith 1918). He described Harvey's approach as “No theory of the function of an organ could be true that did not explain every detail of its structure.” This review attempts to define where we have reached in relating the complex architecture to the function of the cardiac ventricles.

## Historical Background

Modern concepts of cardiac function began with William Harvey who in 1628 explained that blood was expelled from the ventricle by contraction of circular fibers (Leake 1941). He recognized that not all fibers were circular, stating that “all fibers spirally arranged become straight on contraction”. He also observed twisting during systole, describing a “peculiar side‐wise twisting turning toward the right ventricle as if it twists slightly on itself in performing its work”, but there is no evidence that he regarded twisting as functionally important. On the other hand, twisting was regarded as important by Giovanni Borelli (1608–1679) and by Richard Lower (1631–1691) who described spiral fibers and a wringing motion of the heart in systole. Lower (1932) in his 1669 book, Tractatus de Corde, wrote that the ventricle consisted of a “double row of fibers crossing in opposite directions – the outer ones stretching across from left to right and encircling the whole of the parenchyma in their folds, and the deeper ones being carried in the directly opposite direction – and so, since they draw the walls of the Heart more closely together on all sides, the interventricular spaces must necessarily be greatly diminished and constricted. The process can, therefore, not unfittingly be compared with the wringing of a linen cloth to squeeze out the water….

Moreover, some of the fibers of the Heart are straight, but all the others twist round the apex and the whole of its surface in an oblique and contrary direction to end in lines in its base. Hence, these fibers not only compress and (p. 80) diminish the interventricular cavity, whenever they contract on all sides, but they also bring the apex nearer to the base..” This is close to the modern description of cardiac anatomy and physiology.

More details on the development of ideas about cardiac anatomy can be found in the review by Streeter (1979).

Sallin (1969), in an extremely important publication, used simple mechanical principles to show that without spiral fibers the left ventricular ejection fraction could not exceed 41%, but with spiral fibers could be as high as 90%. Ingels et al. (1975) implanted tantalum markers on human hearts to measure midwall dynamics, and commented on ventricular twisting. Arts et al. (1979) showed that twisting helped to equalize transmural differences in sarcomere length and end‐systolic fiber stress. Since then, twisting has been studied intensively.

## Anatomy

Borelli and Lower described spiral fibers, but with little detail, and it was de Senac who in 1749 described that in the left ventricle the subendocardial and subepicardial fibers ran vertically, whereas those in the midwall were circumferential. More detail was added by Pettigrew (1864) who dissected the mammalian heart (as well as the hearts of fish, reptiles and birds). In this remarkable study he wrote:

I. By exercising due care, I have ascertained that the fibres constituting the ventricles are rolled upon each other in such a manner as readily admits of their being separated by dissection into layers or strata, the fibres of each layer being characterized by having a different direction …

III. There is a gradual sequence in the direction of the fibres constituting the layers, whereby they are made gradually to change their course from a nearly vertical direction to a horizontal or transverse one, and from the transverse direction, back again to a nearly vertical one.

Although Pettigrew divided the ventricular wall into seven layers, Streeter et al. (1969) described a continuous change in helix angle from about +90° at the epicardial surface to about zero (circumferential, parallel to the short axis) at the midwall and then to about –90° at the endocardial surface. The pattern of fiber angles was similar in the septum and the free wall of the left ventricle. In the lower apical, third of the left ventricle and just below the semilunar valves (membranous septum) there are no circumferential fibers – perhaps explaining why the apical wall is so thin (Thomas 1957; Streeter et al. 1969). Because the fibers are in the wall of a conical ventricle, those that are oblique form helices. The subepicardial fibers, with an average angle of about 60° to the azimuth, form a left‐handed outer helix (**e**xterior‐l**e**ft); the deeper subendocardial fibers also have an average angle of about −60° and form a right‐handed inner helix (**i**nterior‐r**i**ght). See figure 2 by Sengupta et al. (2008).

**Figure 1:**
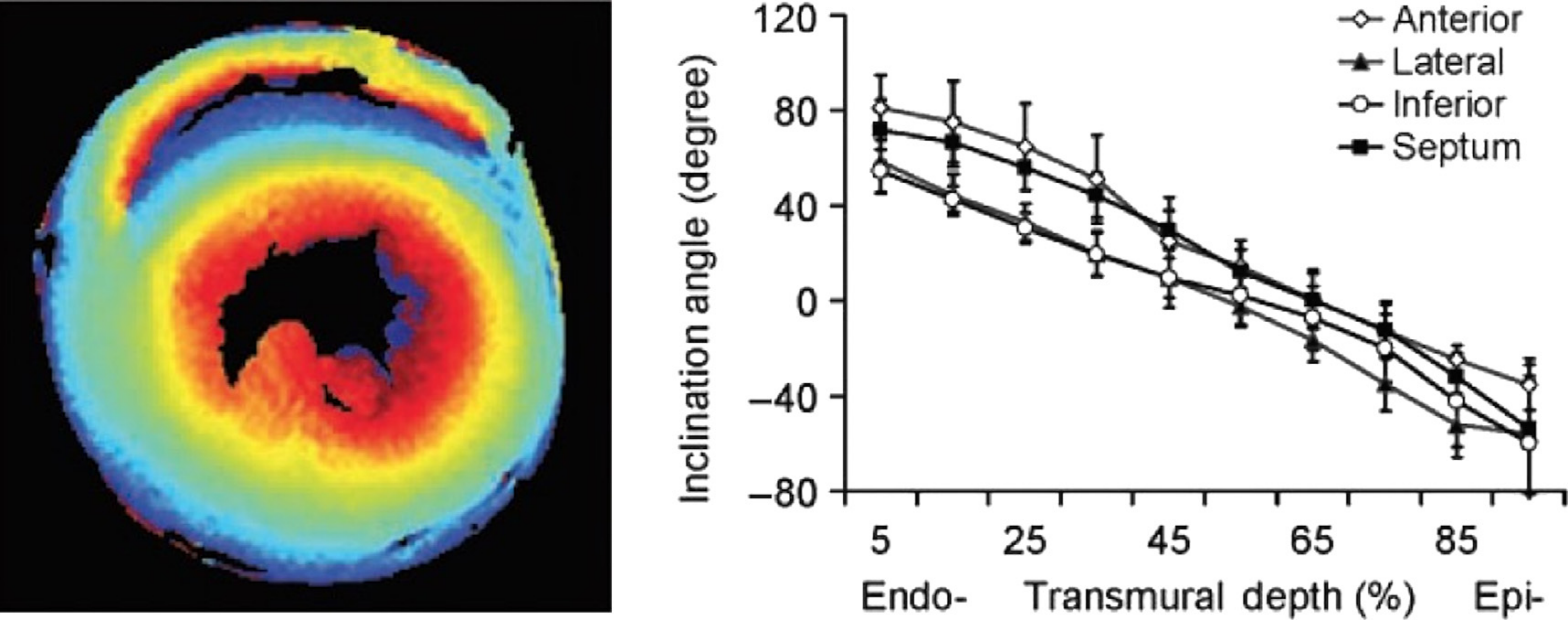
Rat heart. Left panel shows color‐coded helix angles, and right panel shows actual helix angles. These were confirmed by histological measurements. The change in angles is continuous, even though the color‐coding shows sharp transitions. Reproduced with permission from Chen et al. Remodeling of cardiac fiber structure after infarction in rats quantified with diffusion tensor MRI. *Am J Physiol Heart Circ Physiol* 285: H946–954, 2003.

The pattern of fiber angles has been confirmed in mammalian hearts by diffusion tensor MRI (DT‐MRI), a method that relies on the greater mobility of protons in the longitudinal than the lateral direction to detect parallel bundles of fibers (Fig. 1).

Most DT‐MRI studies are usually performed with dead hearts, but the same pattern is seen in living humans, although with lower resolution (Tseng et al. 2000; Wu et al. 2006). These images are based on extensive postprocessing and mathematical manipulation of data. Because of the statistical averaging and the methods used, longitudinal fibers are emphasized and lateral connections are ignored. The fiber angle data obtained by DT‐MRI, polarized light, and histology are similar, (Hsu et al. 1998; Scollan et al. 1998; Jouk et al. 2000) and most studies give a range of angles from 60° to 80° degrees beneath the subepicardium and from −40° to −60° beneath the subendocardium.

Because the HVMB model denies that there are circumferential fibers in the septum, it is important to note that septal circumferential fibers have been described in humans (Greenbaum et al. 1981; Jouk et al. 2000; Tseng et al. 2000; Augenstein et al. 2001; Helm et al. 2005; Wu et al. 2006; Frindel et al. 2010; Eggen et al. 2012; Agger et al. 2016), goats (Geerts et al. 2002), pigs (Thomas 1957; Smerup et al. 2009), dogs (Thomas 1957; Streeter et al. 1969; Hsu et al. 1998), rats, (Hsu et al. 2001; Chen et al. 2003; Hales et al. 2012; Teh et al. 2016a) mice (Jiang et al. 2004; Healy et al. 2011; Sosnovik et al. 2014) sheep (Healy et al. 2011) and rabbits (Scollan et al. 1998; Holmes et al. 2000; Sosnovik et al. 2009; Liu, 2010; Healy et al. 2011; Teh et al. 2016a,b). In other publications, the composition of the septal musculature and free wall of the left ventricle is less clear by DT‐MRI in humans (Zhukov and Barr 2003; Helm et al. 2006; Lombaert et al. 2012; Toussaint et al. 2013; Wei et al. 2013) and pigs (Schmid et al. 2005). Although the preponderance of publications favor distinct circumferential septal layers, DT‐MRI techniques vary considerably from one to another, and the postprocessing that occurs is very complex and also differs between different studies. On the other hand, microscopic studies of fiber angles in dogs (Streeter et al. 1969; Hsu et al. 1998), and rabbits (Scollan et al. 1998; Holmes et al. 2000) are unambiguous, and confirm the findings of most of the DT‐MRI studies. Direct comparisons of fiber angles measured by DT‐MRI and histological preparations showed close agreement (Hsu et al. 1998; Scollan et al. 1998; Holmes et al. 2000).

## Models

Scientific hypotheses are studied using models, either implicitly or explicitly. A model is a representation of reality, but must never be confused with that reality. For example, investigators have constructed models of the sequence of events in angiogenesis, but almost certainly other proteins and chemical agents will be involved, some yet to be discovered. The model allows the investigator to predict what would happen if one section of the sequence is blocked, and devise a suitable experiment. If the prediction is confirmed, then to that extent the model is useful. If it is not confirmed, then the investigator must search for the alternative pathways and modify or alter the model.

Models do not have to be correct to be useful, but usually function within certain limits. If those limits are exceeded the model ceases to be useful. Investigators had long sought a way of assessing myocardial contractility that was independent of preload and afterload. In 1962, Sonnenblick (1962) had a bright idea of measuring *V*_max_, the initial velocity of the unloaded papillary muscle. In a series of elegant studies of the papillary muscle he demonstrated the value of the concept. (Sonnenblick realized that there were different models of muscle function, but was satisfied that his concept was correct.) To use this concept in the whole heart, cardiologists used d*P*/d*t*_max_ for *V*_max_, and in numerous studies it seemed to quantify contractility (as judged by the response to adrenergic stimulation and blockade) independent of preload and afterload. Then, Pollack (1970) published a critical analysis of the subject, and concluded that “inotropic shift of *V*_max_ of the contractile element cannot be distinguished from a shift due to change in fiber length, thus invalidating it as an index of contractility.” Despite further arguments and experiments there was a general perception that Pollack was correct. Therefore, the model as originally constructed was wrong, but that does not mean it was not useful. In practice, d*P*/d*t*_max_ is still a good index of contractility provided there are not large changes in preload and afterload. Furthermore, in the decade following the introduction of the model, the innumerable studies of muscle mechanics advanced the field immensely. What makes a model good is not being correct, but being useful.

## Myocardial Models

There are two general types of myocardial models that relate muscle anatomy to function – microscopic and macroscopic. The microscopic models deal with relations between sarcomeres, and between sarcomeres and connective tissue. For example, LeGrice et al. (1995) found that myofibers clustered in groups (laminae) of 1–12 (average 4) surrounded by collagen. These laminae formed curved sheets that at any given depth from the surface had the same fiber angles as described above. As two‐dimensional structures based on geodesics the individual fibers have the same azimuth angles as if they were flattened sheets. In addition, LeGrice et al. (1995) observed transverse clefts. Fernandez‐Teran and Hurle (1982) showed by microscopy what might be laminae stacked one above the other and enclosed by connective tissue to form longitudinal arrays that are probably the basis for what most investigators call myofibers.

These laminar arrangements allowed myofiber bundles to slide over each other during systole, thereby explaining why wall thickening was more than could be accounted for by fiber thickening alone (Spotnitz et al. 1974; Ingels et al. 1989; LeGrice et al. 1995). They also help to equalize transmural stresses and strains (Arts et al. 1991; Rijcken et al. 1999; Savadjiev et al. 2012). Lunkenheimer et al. (2004) studied the oblique interconnecting fibers with tiny force gauges and observed that in systole the forces increased. They concluded that these oblique myocytes contributed to stiffening of the ventricular wall. Smerup et al. (2013a,b) presented more details about these cross‐connections.

Macroscopic models ignore fine details, and concentrate on the large‐scale distribution of myofibers. There are many such models, and I will concentrate on two of them that may be termed discrete (band) and diffuse (nested layer) models. One much used discrete model is the helical ventricular myocardial band^1^ (HVMB) of Torrent‐Guasp. He first introduced this model in 1966, it was mentioned by Streeter in his 1979 review (Streeter 1979), but little attention was paid to it until a series of articles with Buckberg brought it to the forefront (Torrent‐Guasp et al. 2001a,b). The HVMB was developed by blunt dissection of boiled hearts of several species. From the right ventricle a band of transverse (circumferential) muscle forms the back of the left ventricle; this is the basal transverse loop. From the crest of this loop a sheet of oblique muscle passes down inside the circumferential muscle toward the apex (DS or descending loop), rotates to form a figure 8 at the apical vortex, and then extends up toward the base (AS or ascending loop). This model has been used successfully to explain normal and abnormal cardiac function (Buckberg et al. 2015a,b). The problems with this model are:

1. No anatomic basis has been found for the bands which do not consist of parallel fibers.
2. Blunt dissection disrupts fiber connections.
3. It does not take into account the varied helical angles,
4. It does not explain how the oblique muscle produces both subepicardial and subendocardial helical fibers.
5. It considers the circumferential fibers as enclosing both sets of spiral fibers, whereas they are interposed between the two sets of helical fibers.
6. The model pays no attention to interconnections between fibers, and so has no way of equalizing stresses and strains.
7. The model does not accept circumferential fibers in the septum which is at odds with histological and DT‐MRI evidence.

The nested layer model is more traditional, but has not yet been completely linked to known muscle functions. It considers the left ventricular wall (and septum) as being made of concentric layers of muscle, each with a different helical angle. These layers are connected to each other by transverse components. In 1891, Krehl described such a pattern, called a Triebwerk (an engine or drive mechanism), and the nested interconnected layers described by Streeter and Hanna as concentric ellipsoids of revolution (Streeter and Hanna 1973) match his pattern. In the human fetus, (Jouk et al. 2000) described the myofibers as being geodesics, with helical angles that varied with depth from the surface. Similar nested layers were described in computational models (Peskin 1989; Legrice et al. 1997; Bayer et al. 2012). As shown by Grant (1965) and Anderson et al. (2009), helices with different pitches occur in the same heart; some are almost horizontal with a small pitch and others more vertical with a larger pitch. Some investigators have envisaged the left ventricle as a three‐dimensional continuum (Lunkenheimer et al. 2006; Anderson et al. 2009; Bernus et al. 2015), putting more emphasis on the connecting fibers. The layered model is therefore a simplification of a more complex structure, but considering sheets and connections between sheets separately is more traditional (LeGrice et al. 1995; Costa et al. 1999).

There are many mathematical models of the left ventricle. Many of them begin with a few simple physical principles and then use finite element analysis or Lagrange–Dirichlet algorithms to develop a model that resembles closely what has been found in the heart. For example, Peskin (1989) concluded that the heart consisted of a set of approximate geodesics on a nested family of toroidal surfaces. Others, using different assumptions and mathematical methods, have reached similar conclusions (Legrice et al. 1997; Bayer et al. 2012). An analysis by Savadjiev et al. (2012) concluded that apart from their role in twisting the ventricle during contraction, the helical bundles stiffen the wall, help to equalize forces across the wall, and by forming a minimal surface economize fiber length and optimize ejection volume as they contract.

The vortex of the left ventricle has not been fully described. From the surface it shows a spiral with a central dimple, and some investigators regarded the vortex as the site at which subepicardial muscle invaginated to become subendocardial (Edman and Flitney 1977; Frindel et al. 2010; Bernus et al. 2015; Buckberg et al. 2015a). This idea, however, is difficult to reconcile with the complete but separate helical layers shown in Figure 1. Given that the superficial and deep helical bands cross each other at the apex, one possible anatomical formation is that of a figure 8 at the apex, with the subepicardial helical muscle invaginating, as observed, but continuing as the right ventricular side of the septum while the subendocardial helical muscle continues as the left ventricular side of the septum. A possible mechanism for this rotation during development was described by Manner (2004). Nevertheless, whether the left side of the septum is continuous with the free wall subepicardial or subendocardial muscle has not been established.

Both of these models are oversimplified, and the reality is much more complex (Gilbert et al. 2007).

## Ventricular Function

### Terminology

**Figure 2:**
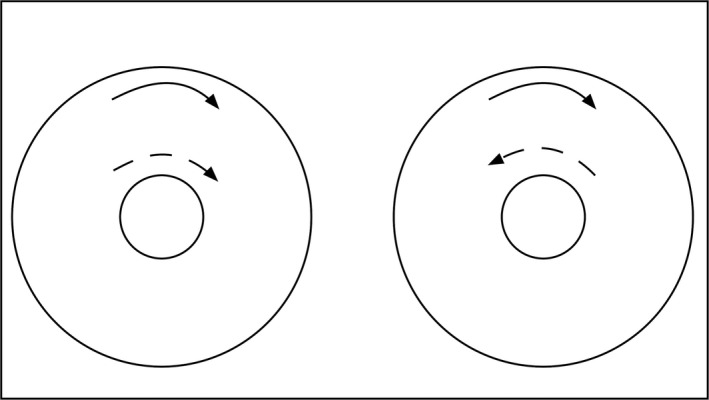
Rotation and twist, as seen looking at the heart from the apex. The larger circle and solid arrow represent the base, and the smaller circle and dashed arrow represent the apex. Left panel: Both apex and base rotate in the same direction; therefore no twist. Right panel: Base rotates clockwise and the apex rotates counterclockwise, producing twist.

Rotation occurs when the heart spins around its long axis (Fig. 2, left). If one part of the heart rotates clockwise and another part rotates counterclockwise, (Fig. 2, right) the difference in rotational angles is termed twist. Some investigators use the terms torsion and twist interchangeably, but currently torsion is defined as rotation per unit length of the LV long axis. It is also possible for twist to be superimposed on global rotation. For example, the whole heart may rotate counterclockwise relative to body by 8° (as shown best by midwall rotation), but the apex may twist 4° clockwise relative to the base so that the apex has rotated counterclockwise by 4° relative to the body. The analogy is a train moving west at 60 mph and a passenger moving east along the corridor at 4 mph. The net movement of the passenger is west at 56 mph.

We now need to explain what motions the left ventricle performs during the cardiac cycle. In functioning as a pump, the left ventricle needs to raise its pressure to overcome the resistance of the aortic blood pressure. The left ventricular muscle does so by squeezing its contained blood, thereby narrowing and lengthening the left ventricular cavity. Once ventricular pressure is high enough to open the aortic valve, blood leaves the ventricle which decreases in volume mainly due to shortening and twisting This reduced length pulls the atrioventricular rings toward the apex, when the ventricle relaxes, its pressure decreases, the aortic valve closes, and rapid untwisting is associated with opening of the mitral valve and then rapid filling of the left ventricle. The atrioventricular valve rings move back toward the base.

To understand how ventricular architecture and function interact, we need to understand that it is not possible to stimulate or inhibit a particular set of fibers to determine how these changes affect cardiac function. Instead we have to use computational models and logic to deduce the function of any set of fibers.

Start with the idealized functions of the different layers. The midwall circumferential layers, with azimuth angles from about −30 to +30°, are constrictors. When they contract they narrow and lengthen the left ventricle, (Rushmer et al. 1953; Rushmer 1956) and by maintaining contraction while left ventricular pressure is high during systole they prevent the left ventricle from ballooning out. The circumferential fibers in the septum are part of the constriction, and they prevent the septum from being pushed toward the right ventricle. (In the HVMB model there would be no counterforce to contraction of the left ventricular free wall, and this contraction would push the septum into the right ventricle and leave it there.) Fibers that encircle the left ventricle more obliquely probably help to prevent overelongation of the left ventricle and the tendency for the elongating ventricle to separate the apex from the base.

The oblique fibers have more complicated functions. When these helical fibers contract they shorten the long axis of the ventricle and also cause the apex to rotate; contraction of the subepicardial fibers rotates the apex counterclockwise, and contraction of the subendocardial fibers rotates the apex clockwise (looking up at the heart from the feet). It is likely that the more horizontal helical fibers are the main causes of torsion, whereas the more vertical helical fibers play a more important role in shortening. Which way the apex rotates depends on the balance of these two forces (see below). These descriptions are oversimplified; interconnections between muscle layers indicate that these movements are not independent of each other.

**Figure 3:**
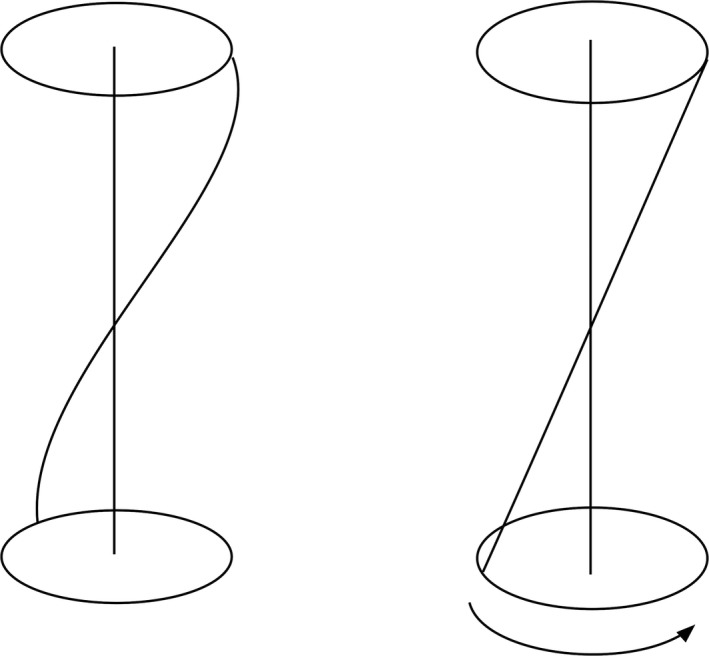
Activation of helical fibers when the two ends are fixed. There will be twist but the distance between the two ends remains the same.

Although activation of the myosin‐actin cross‐bridges of the helical fibers usually results in shortening and rotation, under certain constraints these two motions are dissociated. (By activation, I mean the process from release of calcium from the sarcoplasmic reticulum to the generation of force by actin and myosin.) If the tendency to shorten is opposed by a force in the opposite direction, the two ends of the helical fibers may not move toward each other or may even move apart. Myocyte contraction will, however, tend to straighten out the helix and cause some twist (Fig. 3).

Another distinction between the two models is that the HVMB model does not take the numerous connections between fiber layers into account. Usually, when a muscle is activated, and the fiber gets shorter. With force inactivation, calcium is returned to the sarcoplasmic reticulum, actin and myosin cease to interact, and rebound of titin springs restores the myofiber to its resting length. When myofibers in one layer are connected to myofibers in other layers, however, more complex interactions occur. Despite actin–myosin interaction a muscle may lengthen, not shorten, if an external force counteracts the tendency to shorten, as occurs in subepicardial muscle in the presystolic isovolumic period. Alternatively, after uncoupling actin and myosin a muscle might not lengthen if it is connected to a muscle in an adjacent layer that is still contracting. Measuring length alone, as with a sonomicrometer, will not distinguish between activation and inactivation. Some simultaneous measurement of force must be added so that the correct conclusion will be drawn.

**Figure 4:**
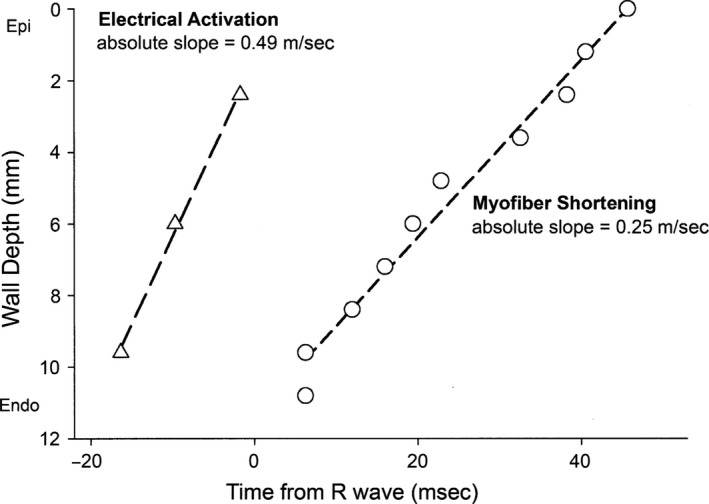
Electrical activation and shortening times. Reproduced with permission from Ashikaga et al. Transmural dispersion of myofiber mechanics: implications for electrical heterogeneity in vivo. *J Am Coll Cardiol* 49: 909–916, 2007.

One other distinction between the two models is that Ashikaga et al. (2007) demonstrated that despite rapid electrical activation from endocardium to epicardium, there was much slower spread of shortening (Fig. 4). This is similar to the dispersion shown in a different model by Sengupta et al. (2007).

It took about 40–50 msec for shortening to occur in the subepicardium after it had begun in the subendocardium, and in fact there was initial lengthening of the subepicardial muscle before it began to shorten, as described by Sengupta et al. (2005). This apparent paradox occurs because although the subepicardial muscle is activated, it is lengthened by the greater force exerted by the elongating ventricle and the torsion produced by the subendocardial helix. An analogy is if you contract your biceps but someone forcefully extends it while you maintain tension. This also implies that delayed shortening after activation ends may be due to balanced opposing forces. The ripple effect of transmural activation does not support the HVMB model.

#### Presystolic isovolumic contraction

At the onset of systole the subendocardial right‐handed helical fibers are stimulated to contract by the impulses from the Purkinje system, causing transient shortening of the left ventricle and transient clockwise rotation. These are followed a few milliseconds later by contraction of the midwall circumferential fibers that exert greater force than the inner fibers because of their greater mass and greater moment arm, so the net effect is to narrow and elongate the ventricle. Boettler et al. (2005) and Hayabuchi et al. (2015) observed that systolic strains were greater in the inner half of the septum and began earlier there than in the outer half, as expected from the endocardial to epicardial direction of activation. This elongation actually stretches the subepicardial left‐handed helical fibers (Sengupta et al. 2005, 2006, 2008; Ashikaga et al. 2007). Velocity vector imaging (VVI) shows that in systole the upper part of the septum (the membranous septum) which does not have circumferential muscle balloons into the right ventricle during isovolumic contraction. There is transient movement of the lower septum toward the right ventricle, but this is reversed when the circumferential muscle contracts, thereby allowing the left ventricle to narrow and its cavity pressure to rise. In addition, there is global counterclockwise rotation (Jung et al. 2006; Russel et al. 2008). The mechanism is not clear; one possibility is that the subepicardial muscle produces counterclockwise rotation of the midwall and apex, but does not cause twisting because the subendocardial helical fibers prevent clockwise rotation of the base.

There is slight twisting of the apex and base, but the descriptions are variable, depending on methodology. By tagged MRI, four studies all showed slight counterclockwise rotation at the apex, and two showed slight clockwise rotation at the base. By echocardiography, apical rotation was counterclockwise in eight, clockwise in four, and basal rotation was clockwise in nine, and counterclockwise in three. Results differ in part because the measurements may be made at different distances from the apex, and because sometimes only the subepicardium is imaged. The MRI results may be more reliable because they always include the whole wall. The twist during this period is slight and probably of little functional importance, but the methodological differences will cause variability when assessing normal twist values during ejection.

#### Systole

**Figure 5:**
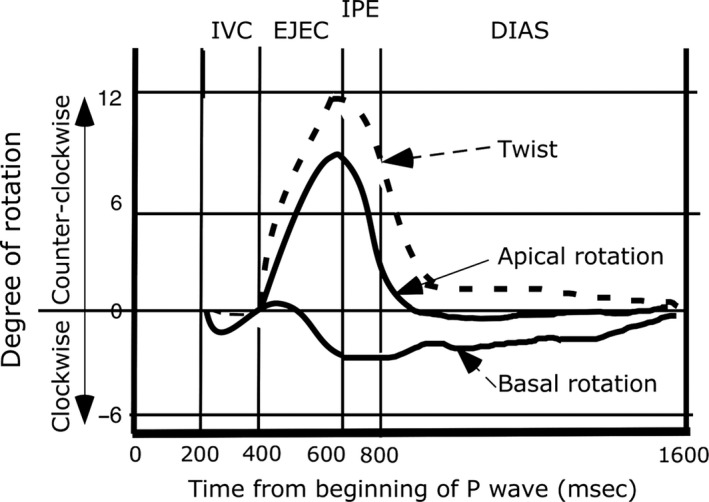
Twist diagram. IVC‐isovolumic preejection period; EJEC‐ejection; IPE‐isovolumic postejection period; DIAS‐mid and ‐late diastole.

The circumferential fibers remain contracted, and the contracting helical fibers help to expel blood from the left ventricle, causing descent of the base toward the apex. Now the rotational capabilities of the two sets of helical fibers come into play. Because the subepicardial helical fibers have the larger moments they exert greater torque than do the subendocardial fibers, and dominate the apical rotation which moves counterclockwise. In addition, there is a gradient of myosin RLC phosphorylation in the left ventricular free wall that favors contractility in the subepicardial muscle (Davis et al. 2001). At the same time there is a slight clockwise rotation of the base which is less free to move than the apex. Consequently, the left ventricle twists. A typical twist diagram is shown in Figure 5.

#### Postejection isovolumic period

This is the most complicated and least understood part of the cardiac cycle. It used to be termed the isovolumic relaxation period, but this is a misnomer. The period is indeed isovolumic, but not all of the left ventricular muscle is relaxing. The continued contraction of some muscle after ejection has ended has been known for a long time, and termed postsystolic shortening. There is slight elongation and widening of the left ventricle, but left ventricular volume is kept constant by movement of the closed mitral valve leaflets toward the apex (Cheng‐Baron et al. 2010) and by reciprocal shortening and elongation of apical and basal portions of the two helical muscles (Sengupta et al. 2007).

Having twisted in systole, the heart must untwist to return to its resting configuration. The untwisting must initially be rapid so that ventricular suction can be initiated (see below). This clockwise rotation of the apex must logically be due to inactivation of the left‐handed subepicardial helix with continued contraction of the right‐handed helix. Delayed relaxation of the left‐handed helix or inadequate contraction of the right‐handed helix will delay the onset of untwisting, as shown for aortic stenosis by Stuber et al. (1999). This delay prevents rapid lowering of left ventricular diastolic pressure, and leads to decreased rate of filling of the left ventricle in early diastole, signs of diastolic dysfunction that, as pointed out by Tan et al. (2009) must originate in systole. Inasmuch as subendocardial muscle is at risk of ischemia in many forms of cardiac disease, it is reasonable to look for the cause of delayed untwisting in the subendocardial helix, but there are no studies to prove this.

Many contradictory studies have been published. Several studies have reported that the subendocardial muscle (right‐handed helix) lengthens about 60–80 msec (the hiatus) before the subepicardial muscle (left‐handed helix) begins to lengthen in the left ventricular free wall (Sengupta et al. 2006; Buckberg et al. 2008; Hayabuchi et al. 2015) and for the septum (Hristov et al. 2006; Holland et al. 2010) in which lengthening begins first in the left ventricular side (right‐handed helix). This lengthening begins before aortic valve closure. (This is not surprising, because we know that left ventricular muscle stops generating force about half way through systole; Noble (1968) found that aortic pressure exceeded left ventricular pressure in the second half of systole, and that occluding the aorta in the last third of systole caused a fall in left ventricular pressure. The blood flows from left ventricle to aorta by momentum late in systole). Ashikaga et al. (2007) and Hasegawa et al. (2009) found the opposite – the only conflicting reports – with subepicardial muscle relaxing first; the total delay between subepicardial and subendocardial muscle relaxation averaged 93 msec.

One possible way of reconciling these disparate findings is found in the studies by Sengupta et al. (2007, 2007) who observed that in early diastole the left ventricular subepicardial muscle lengthens at the base while that at the apex shortens, and in subendocardial muscle, the fibers lengthen at the apex and shorten at the base.^2^

It is a mistake to consider the left ventricle as isotropic. There are differences in activation times in different regions (Sengupta et al. 2006) and probably in the order of contraction that depends in part on connections to surrounding layers, and the nested shell concept is a simplification of a more complex anatomy (Smerup et al., 2013b). Relaxation is a not only a function of movement of calcium back into its storage sites but also of load dependence (Brutsaert et al. 1980). Left ventricular strains are heterogeneous (Bachner‐Hinenzon et al. 2010; Leitman et al. 2010), so that it is likely that load dependence effects on relaxation differ in different parts of the left ventricle. In fact, even in striated skeletal muscle, during relaxation it is possible to find that some sarcomeres are getting shorter while others are getting longer (Edman and Flitney 1977).

With the exception of the studies by Ashikaga et al. (2007) and Schmid et al. (2005), other studies of contraction across the left ventricular free wall or septum have used two regions of interest, one related to the right‐handed helix and one to the left‐handed helix. What might help decide between the two models is having more regions of interest across the wall's thickness, and making measurements at different distances from the apex. If the nested model is correct, there should be a dispersion of contraction and relaxation times in different layers and perhaps different distances from the apex. On the other hand, finding one set of times in the inner layer and another set in the outer layer would support the HVMB model.

The left ventricular cavity widens due to relaxation of circumferential muscle and rebound of the compressed titin springs (Rushmer et al. 1953; Suga et al. 1986; Gilbert and Glantz 1989). It also lengthens slightly prior to filling with blood, and this is probably due to relaxation of some of the subendocardial muscle (right‐handed helix) and some of the subepicardial muscle, and rebound of their titin springs, because the some of the left‐handed helix (subepicardial muscle) is still contracted. The apex starts to rotate clockwise under the unopposed influence of the right‐handed helix. Figure 5 shows that normally untwisting begins just before aortic valve closure. At the same time, there is global clockwise rotation (Jung et al. 2006).

The lengthening and widening of the left ventricle cause the pressure in the left ventricle to decrease or even in some circumstances become negative, and once the mitral valve is open this decreased pressure allows atrial blood to be sucked rapidly into the ventricle.^3^ It is the decreased left ventricular early diastolic pressure and not the opening of the mitral valve that is important for rapid filling early in diastole, because the mitral valve opens 15–45 msec before blood starts to move from left atrium to left ventricle (Lee et al. 1990; Karwatowski et al. 1996).

If the subendocardial muscle fails to relax early, then there is less twist because the left‐handed helical movement is no longer unopposed, the left ventricle does not lengthen, low or negative left ventricular early diastolic pressures are not generated, and early filling is slowed; these are all features of diastolic dysfunction.

##### Late diastole

Late diastolic filling occurs, aided by atrial contraction. The left‐handed helix relaxes, and the heart untwists to return to its resting position, ready for the next cycle.

##### Right ventricle

The right ventricle has a thin free wall. Originally, the wall was described as having mainly circumferential fibers (Ho and Nihoyannopoulos 2006). One study with DT‐MRI confirmed this (Hsu et al. 2001), but three other such studies described three layers, as in the free wall of the left ventricle (Scollan et al. 1998; Geerts et al. 2002; Chen et al. 2003). More recently studies have described predominantly oblique fibers with very sparse circumferential fibers (Nielsen et al. 2009). However, most of right ventricular ejection with normal pulmonary arterial pressure is due to left ventricular forces, in particular the contraction of the thicker septal helical muscles. The descent of the tricuspid valve ring in systole (TAPSE) with resultant shortening of the long axis of the right ventricle is due mainly to septal contraction and has little to do with the free wall which can be removed or disabled without detriment (Kagan 1952; Donald and Essex 1954; Sawatani et al. 1974; Cox et al. 1985; Yamaguchi et al. 1993; Yaku et al. 1994).

## Conclusions

The contributions of Torrent‐Guasp and his HMVB model cannot be overestimated. Despite 17th century studies emphasizing the importance of twisting, physiologists, clinicians lost sight of it, probably because most cardiac investigations by angiography or echocardiography involved two‐dimensional images. It was Torrent‐Guasp's description of oblique fibers, supplemented by the enthusiastic support of Buckberg at UCLA and Sengupta at the Mayo Clinic, that emphasized twisting as a vital part of cardiac function. Both the band and nested models can explain much of how the heart functions during the cardiac cycle and how function is affected by disease. This is not unexpected because both models incorporate circumferential fibers that narrow the ventricle in systole, and helical fibers that in systole shorten and rotate the left ventricle. The distributed model is preferred, however, because it takes account of circumferential fibers and cross‐connections that are ignored by the HVMB model.

Further studies are required to clarify some of the paradoxes described above. What factors are responsible for elongation of part of a muscle and shortening of another part of the same muscle? Can heterogeneity of contraction be explained by tethering, or are there heterogeneities of biochemical activity that must be accounted for?

In time, better models will be introduced. These will need to take account of the irregularities of the anatomy as well as differences in biochemistry in different regions of the left ventricle. Nevertheless, the present simplified model is compatible with known anatomy and accounts for ventricular function over the cardiac cycle and allows prediction of what occurs during disease.

## Conflict of Interest

None declared

## Notes

1 The term “band” has not been rigorously defined. (Torrent‐Guasp et al. 2001b) wrote: “When we describe fibers, fascicles, or bands of myocardium, we do not intend that these are specific anatomic structures that are exactly observed with precision in different hearts. A selected muscular mass shows certain preferred trajectories visualized along the longitudinal axis of any muscular strip.”

2 The disparate effects in the early postejection period (lengthening at the apex and shortening at the base) are not easily explained. When cardiac muscle fibers are deactivated and the actin‐myosin cross‐bridges disconnect, the fibers usually lengthen due to rebound of compressed titin springs. However, the fibers are also affected by external forces. At the base, continued contraction of the outer helical muscle might by tethering prevent the inner helical muscle from lengthening. Why this does not occur at the apex is unexplained, but the absence of circumferential fibers near the apex is at least one anatomic difference that might alter tethering.

3 Several studies in dogs and humans have shown very low pressures in the left ventricular cavity in early diastole, as well as pressure differences within the cavity and between the left ventricle and left atrium (Falsetti et al. 1980; Courtois et al. 1988; Nikolic et al. 1995; Guerra et al. 2013).
